# Thermal Conductivity of Polybutadiene Rubber from Molecular Dynamics Simulations and Measurements by the Heat Flow Meter Method

**DOI:** 10.3390/ma14247737

**Published:** 2021-12-15

**Authors:** Aleksandr Vasilev, Tommy Lorenz, Vikram G Kamble, Sven Wießner, Cornelia Breitkopf

**Affiliations:** 1 Chair of Technical Thermodynamics, Technische Universität Dresden, 01069 Dresden, Germany; tommy.lorenz@tu-dresden.de (T.L.); cornelia.breitkopf@tu-dresden.de (C.B.); 2 Leibniz-Institut für Polymerforschung Dresden e.V., Hohe Str. 6, 01069 Dresden, Germany; kamble@ipfdd.de (V.G.K.); wiessner@ipfdd.de (S.W.)

**Keywords:** molecular dynamics simulations, force field, rubber, polybutadiene, thermal conductivity, heat flow meter method

## Abstract

Thermal conductivities of polybutadiene rubbers crosslinked by 2.4 and 2.8 phr of sulfur have been found to be functions of temperature via molecular dynamics (MD) simulations using the Green–Kubo method. From an analysis of the heat flux autocorrelation functions, it has been revealed that the dominant means of heat transport in rubbers is governed by deformations of polymeric chains. Thermal conductivities of rubber samples vulcanized by 2.4 and 2.8 phr of sulfur have been measured by the heat flow meter method between 0 ${}^{\circ }$C and 60 ${}^{\circ }$C at atmospheric pressure. The temperature dependencies of the thermal conductivities of rubbers and their glass transition temperatures derived from MD simulations are in good agreement with the literature and experimental data. Details are discussed in the paper.

## 1. Introduction

Rubbers are widely used for the development of new composite materials [1,2]. To predict the behaviour of composites under different conditions, modelings are conducted using the finite element method (FEM) [3,4]. Conservative equations are solved by FEM for each composite element. These equations require knowledge of material properties, for instance, thermal conductivity.

Knowledge of the thermal conductivity is important not only for the modeling of rubber-based composites, but also for enhancement of rubber injection molding processes [5]. The thermal conductivity of rubber can be determined by theoretical approaches and experimental techniques.

The transient hot wire method is widely used for measurements of thermal conductivities of rubbers and rubber composites [6,7,8]. The method is based on measuring the temperature change at some distance from the wire, which acts as a heat source and is embedded into a sample. The sample has to be isotropic. This method is faster than steady state techniques and it is an absolute method. There are standards for measurements, such as ASTM C 1113, ISO 8894-1 and ISO 8894-2.

Another transient method for measurements of thermal conductivities of rubbers and composites is the Laser Flash technique [5,9,10,11,12,13,14]. In this method the thermal diffusivity of a sample is measured, therefore it requires knowledge of heat capacity and density to find the thermal conductivity.

For materials with low thermal conductivity, such as rubber, the guarded hot plate [8,15,16,17] and heat flow meter methods [5,18,19] are often used. These techniques belongs to steady state approaches, as a result, require more time for measurements in comparison with transient methods. The guarded hot plate method is an absolute technique of measurement and is more accurate for insulators than the Laser Flash technique.

In Ref. [5] thermal conductivities of industrial rubber compounds obtained via the Guarded Heat Flow Meter, Laser Flash, Plane-Source and Line-Source techniques have been compared. The Guarded Heat Flow Meter and the Laser Flash techniques provide comparable results. It was revealed, that thermal conductivity of unfilled natural rubber remains almost constant from 60 ${}^{\circ }$C until 180 ${}^{\circ }$C.

In other research [20] it was observed that the thermal conductivity of rubbers varies slightly with temperature, reaches a global maximum at the glass transition temperature and then decreases. On the other hand, for some rubbers, such as natural rubber, polyisobutylene, soft and hard rubber at temperatures above room temperature, it remains almost constant [5,20,21]. More investigations are needed on the thermal conductivity’s dependence on the temperature.

MD simulations can be an alternative tool for determination of the thermal conductivity as function of temperature. For instance, thermal conductivities of untreated polyisoprene and polybutadiene, obtained via MD simulations and measurements by the transient hot wire method, have been compared in Ref. [22]. The results from the simulations are in good agreement with the experimental data. The effect of the composition ratio of styrene on the thermal conductivity of butadiene styrene rubber has been found [23]. In Ref. [24], this has been calculated for soft and hard rubbers, but the difference between the results from simulations and experiments was roughly twice this. There is a great need to develop and test force fields for the prediction of the thermal conductivities of rubbers.

The study intends to compare samples, which have been exclusively prepared using experimental and theoretical methods. Special attention was paid to a detailed crosslinking of the polymers.

## 2. Simulation and Experimental Details

In the first part of the section, simulation details are explained and, in the second part, preparation details are given.

### 2.1. Simulation Details and Description of the Experimental Setup for Measurement of the Thermal Conductivity

Polybutadiene chains for the modeling of rubbers crosslinked by 2.4 and 2.8 phr (parts per hundred rubber) of sulfur have been constructed by Moltemplate software [25]. The chains consisted of 212 and 200 monomer units. A united atom force field description was used, where CH, CH${}_{2}$, and CH${}_{3}$ groups were modeled as one united atom; thus, carbon and hydrogen atoms have not explicitly been treated. The type of crosslink bridge considered in the research was the same as in Refs. [24,26]. The total potential energy of a polymeric system is calculated as

(1) $$E=E_{\mathrm{bond}}+E_{\mathrm{angle}}+E_{\mathrm{dihedral}}+E_{\mathrm{non}-\mathrm{bonded}}.$$

For MD simulations of the rubbers, the same force field as in Ref. [26] was used, where intra-molecular interactions are described by harmonic potentials

(2) $$E_{\mathrm{bond}}=\frac{K_{\mathrm{bond}}}{2}{\left(r-r_{0}\right)}^{2}$$

(3) $$E_{\mathrm{angle}}=\frac{K_{\mathrm{angle}}}{2}{\left(\theta -\theta_{0}\right)}^{2}$$

(4) $$E_{\mathrm{dihedral}}=\sum_{j=1}^{3}\left(\frac{{K_{\mathrm{dihedral}}}_{j}}{2}\left[1+{\left(-1\right)}^{j+1}\cos j\phi \right]\right)$$

where $K_{\mathrm{bond}}$, $K_{\mathrm{angle}}$ and $K_{\mathrm{dihedral}}$ are force constants for bond, angle and dihedral interactions, respectively; *r* and $r_{0}$ are the bond length and the equilibrium bond length, respectively; *r* and $r_{0}$ are the bond length and the equilibrium bond length, respectively; θ and $\theta_{0}$ are the angle and equilibrium angle, respectively; ϕ is the dihedral angle.

The inter-molecular interactions were only presented by van der Waals forces, because CH, CH${}_{2}$, and CH${}_{3}$ groups have a total charge of zero; therefore, Coulomb interactions were not included in the mathematical model. The van der Waals forces were described by the Lennard–Jones potential, which is given by

(5) $$E_{\mathrm{LJ}}=\left\{\begin{array}{cc} 4\epsilon {\left[{\left(\frac{\sigma }{r}\right)}^{12}-(\frac{\sigma }{r}\right)}^{6}] & \mathrm{if}\;r\leq r_{c},\\ 0 & \mathrm{otherwise}. \end{array}\right.$$

where ϵ is the potential energy well depth, σ is the distance between particles, when the Lennard–Jones potential for the particles is equal to zero and $r_{c}$ is a cutoff distance, which was equal to 10 Å in all simulations. The Lorentz–Berthelot mixing rules were used to find the missing parameters of the Lennard–Jones potential for the description of van der Waals forces between atoms of a different type. All simulations were carried out using the LAMMPS [27] software package.

**Figure 1:**
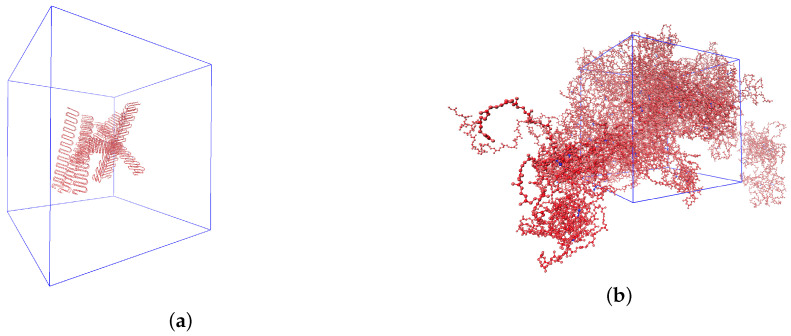
(**a**) Randomly distributed 16 polybutadiene chains in the periodic supercell (size of the cell is 440 Å × 440 Å × 440 Å); (**b**) model of polybutadiene rubber vulcanized by 2.8 phr of sulfur in the periodic supercell (size of the cell is 69 Å × 69 Å × 69 Å).

Firstly, polymeric chains were randomly distributed in a periodic supercell (see Figure 1a). After that, they were crosslinked in a NVT ensemble using a similar algorithm to that of Ref. [28]. Then, high pressure (1000 atm) and temperature (900 K) were slowly, cooly applied to the obtained model of crosslinked chains until normal conditions were reached. This procedure was repeated several times until the density of the model reached a density close to the experimental density. Then, the system was modeled in an NPT ensemble for 100 ps with a time step 1 fs to equilibrate its density. In the next step, it was simulated 100 ps with the time step 1 fs in a NVT ensemble for equilibration of energy and temperature. Finally, when the system was placed in thermal equilibrium in an NVE ensemble, for every 3 ns with a time step 1 fs, the thermal conductivity of the system was calculated via the Green–Kubo formula for every correlation time interval, which was equal to 3 ps. For determination of the thermal conductivity, results from the last 50 correlation time intervals were used to find the mean value. According to the Green–Kubo formula, the thermal conductivity of isotropic material can be found as follows:

(6) $$\lambda =\frac{V}{3k_{B}T^{2}}\int_{0}^{\infty }\;<\vec{J}\left(0\right)\vec{J}\left(t\right)>dt,$$

where $\vec{J}$ is the heat flux calculated by the following equation taken from Ref. [29]:

(7) $$\vec{J}=\frac{1}{V}\left[\sum_{i}e_{i}\vec{\upsilon }-\sum_{i}S_{i}\vec{\upsilon }\right],$$

where $e_{i}$ is the total energy of the *i*-th atom. The first term is the convectional part of the total heat flux, which represents the heat flux due to the movement of atoms in the system. $S_{i}$ is the per-atom stress tensor calculated by the equation [30]:

(8) $$S_{ab}=-m\upsilon_{a}\upsilon_{b}-W_{ab},$$

where *a* and *b* take on the values *x*, *y*, and *z*, and $W_{ab}$ is the virial contribution, calculated as [30]:

(9) $$W_{ab}=\sum_{n=1}^{N_{p}}r_{I0_{a}}F_{I_{b}}+\sum_{n=1}^{N_{b}}r_{I0_{a}}F_{I_{b}}+\sum_{n=1}^{N_{a}}r_{I0_{a}}F_{I_{b}}+\sum_{n=1}^{N_{d}}r_{I0_{a}}F_{I_{b}}+\sum_{n=1}^{N_{i}}r_{I0_{a}}F_{I_{b}},$$

where $N_{p}$ is the number of neighbors of atom *I* that act on atom *I* via van der Waals interaction, and $N_{b}$, $N_{a}$, $N_{d}$, and $N_{i}$ are the numbers of bonds, angles, dihedrals, and impropers, respectively, and the atom *I* is included in these interactions. $F_{I}$ is the force acting on atom *I* due to these interactions, and $r_{I0}$ is the relative position of the atom *I* with respect to the geometric center of the interacting atoms.

Due to the discretization of time in MD simulations, Equation (7) can be written as follows [31]:

(10) $$\lambda \left(\tau_{M}\right)=\frac{V\Delta t}{3k_{B}T^{2}}\sum_{m=1}^{M}\frac{1}{\left(N-m\right)}\sum_{n=1}^{N-m}J_{i}\left(n\right)J_{j}\left(m+n\right),$$

where $\lambda (\tau_{M})$ is the thermal conductivity obtained from summation to time step *M* (*M* = 0, 1, …, *N* − 1), *N* is the total number of simulation steps, and $\tau_{M}$ = *M*Δt.

To investigate the influence of size effects on the thermal conductivity, the results for systems of untreated polybutadiene with 12,000 and 24,000 united atoms were compared. For the system with 12,000 united atoms (size of the cell 66 Å × 66 Å × 66 Å) the thermal conductivity was equal to 0.199 W/m/K, whereas for the bigger system (24,000 united atoms, size of the cell 83 Å × 83 Å × 83 Å) the thermal conductivity was 0.201 W/m/K. For MD simulations of rubbers crosslinked with 2.4 phr and 2.8 phr of sulfur, models with 14,352 (size of the cell 70 Å × 70 Å × 70 Å) and 13,728 (size of the cell 69 Å × 69 Å × 69 Å) united atoms were used (see Figure 1b), respectively.

**Figure 2:**
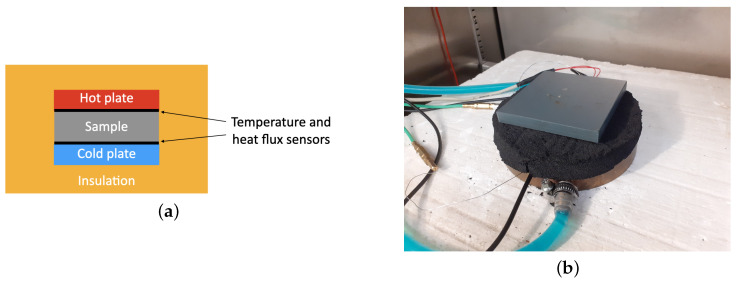
(**a**) Scheme of Heat Flow Meter method; (**b**) experimental setup.

The measurement of the thermal conductivity of rubber samples was carried out using the heat flow meter method. This approach is based on obtaining a constant heat flux and temperature gradient inside a sample. When this condition is achieved, Fourier’s law of thermal conduction can be used to calculate the thermal conductivity of the sample

(11) $$\lambda_{z}=-\frac{{\vec{q}}_{z}}{\frac{dT}{dz}}$$

where ${\vec{q}}_{z}$ is the heat flux in *z* direction, which was taken as the average value of heat fluxes measured by upper- and lower-heat-flux sensors (see Figure 2a), dT is the temperature difference between hot and cold plates and dz is the distance between the plates.

Schematic description of the method is presented in Figure 2a. The sample is placed between hot and cold plates. The upper surface of the sample is heated by a foil connected to a cooper plate. Constant current and voltage were applied to maintain the temperature of the hot plate. Between the plate and the sample temperature, the heat flux sensor was established. Similarly, temperature and heat flux sensors were set up between the bottom surface of the sample and cold plate. The maximal uncertainty of the heat flux sensor was no more than 5%. The temperature of the cold plate was maintained by thermostat. The experimental set up used for measurements is shown in Figure 2b.

### 2.2. Preparation of Samples for Experiments

**Figure 3:**
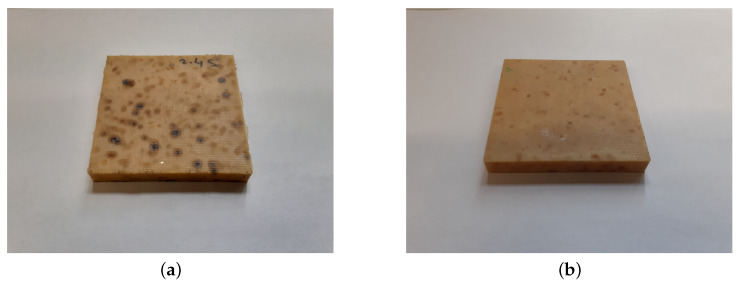
(**a**) Sample of polybutadiene rubber crosslinked by 2.4 phr of sulfur; (**b**) sample of polybutadiene rubber crosslinked by 2.8 phr of sulfur.

To obtain an experimentally equivalent sample basis, new liquid polybutadiene rubber (LBR)-based elastomers were prepared (see Figure 3). A commercially available high-cis LBR-homopolymer (1,2-vinyl content below 5 mol %) with a molecular weight of 45 kDa (LBR-300 by Kuraray Europe GmbH, Hattersheim, Germany) was selected as raw polymer. The crosslinking of the rubber was based on an accelerated sulfur vulcanization system. Sulfur (purity ∼99.5 %) and Zinc Oxide (purity ∼99.5 %) were obtained from Acros Organics N.V. (Geel, Belgium) and used as received. A general purpose grade Stearic acid was purchased from Fisher Scientific GmbH (Schwerte, Germany). The accelerators N-cyclohexyl-2-benzothiazole sulfenamide (CBS) and Tetrabenzylthiuramdisulfide (TBzTD) were obtained from Rhein Chemie GmbH (Mannheim, Germany) and Avokal Heller GmbH (Wuppertal, Germany), respectively. To achieve two different crosslink densities of the vulcanizates, the sulfur content was varied. The formulations are shown in Table 1. All quantities refer to parts per hundred rubber (phr).

The compositions shown in Table 1 were mixed in a scale of 50 g at a temperature of 25 ${}^{\circ }$C using a liquid mixer working with the dual asymmetric centrifuge principal (SpeedMixer, DAC150 SP by Hauschild GmbH & Co KG, Hamm, Germany) at a defined mixing sequence as follows: 800 rpm (5 s), 2500 rpm (120 s), 1200 rpm (5 s), 2500 rpm (100 s), 800 rpm (5 s) and this cycle was repeated three times to ensure the homogeneity of the mixture. Then, the formulations were transferred to a metal mold with rectangular cavities (50 mm × 50 mm × 10 mm and 100 mm × 100 mm × 2 mm) and allowed to cure to their respective T90 time in a compression molding machine (Model TP1000 by Fontijne Presses B.V., Delft, The Netherlands) at a temperature of 150 ${}^{\circ }$C and a force of 150 kN.

## 3. Results and Discussion

**Figure 4:**
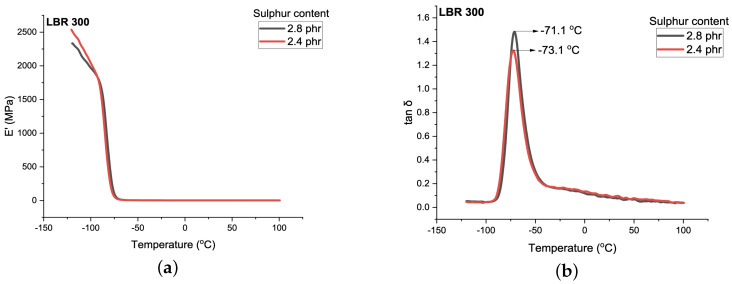
(**a**) Storage modulus ($E^{’}$); (**b**) mechanical loss factor (tanδ) as a function of temperature.

The dynamic mechanical behavior of rectangular samples (30 mm × 10 mm × 2 mm) was characterized by temperature sweep experiments using a mechanical spectrometer (GABO EPLEXOR 150N, GABO QUALIMETER Testanlagen GmbH, Ahlstedt, Germany) in tension mode at 0.5 % dynamic strain amplitude, 2 K/min heating rate (temperature range of −120 ${}^{\circ }$C to 100 ${}^{\circ }$C) and 10 Hz frequency. The storage modulus ($E^{\prime }$) and mechanical loss factor (tanδ) are shown in Figure 4.

From the local maximum of the mechanical loss factor (see Figure 4) the glass transition temperatures $T_{g,\;\mathrm{DMA}}$ can be estimated with ∼−71 ${}^{\circ }$C…−73 ${}^{\circ }$C, indicating a slight shift in $T_{g,\;\mathrm{DMA}}$ towards higher temperatures at an increasing degree of crosslinking, as already known from the literature [32]. This is in agreement with the published data on the glass transition temperature of polybutadiene rubbers with a different microstructure [33]. In this context, it must be noted that the glass transition temperatures obtained by dynamic scanning calorimetry (DSC) are usually ∼20 K lower than those obtained from DMA method [34].

**Figure 5:**
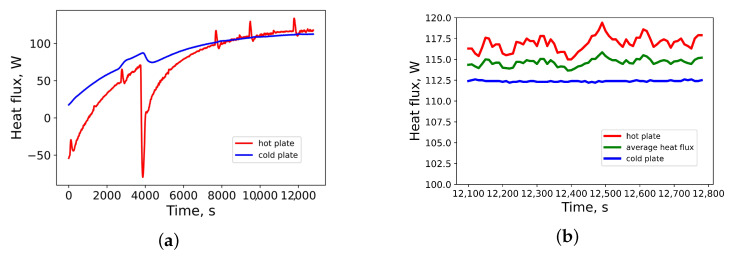
(**a**) Heat fluxes through hot and cold plates; (**b**) heat fluxes in steady-state regime.

Heat fluxes through the upper and lower surfaces of the sample of polybutadiene rubber vulcanized by 2.4 phr of sulfur are presented in Figure 5a. For calculation of the thermal conductivity, only datapoints from steady-state regime were taken. The heat fluxes in the steady-state regime are presented in Figure 5b.

**Figure 6:**
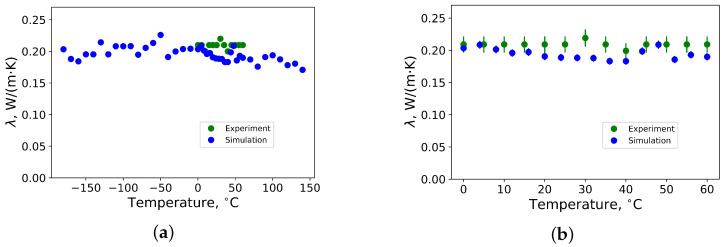
(**a**) Thermal conductivity of polybutadiene rubber vulcanized by 2.4 phr of sulfur in temperature range between −180 ${}^{\circ }$C and 140 ${}^{\circ }$C from MD simulations; (**b**) thermal conductivity of polybutadiene rubber vulcanized by 2.4 phr of sulfur in temperature range between 0 ${}^{\circ }$C and 60 ${}^{\circ }$C from MD simulations (blue) and measurements (green) by the Heat Flow Meter method.

The thermal conductivity of polybutadiene rubber crosslinked by 2.4 phr of sulfur as function of temperature is shown in Figure 6. Standard deviation of results from MD simulations is below 3 %, whereas the deviation of measurements is 6 %. From Figure 6a), that thermal conductivity increases until around −50 ${}^{\circ }$C degrees and, above this temperature, it decreases until it reaches room temperature. This means that the glass transition temperature of the rubber is close to −50 ${}^{\circ }$C degrees, which is in good agreement with the data from measurements (see Figure 4). At temperatures above room temperature, the thermal conductivity slightly decreases. This is in agreement with the results from Ref. [20].

**Figure 7:**
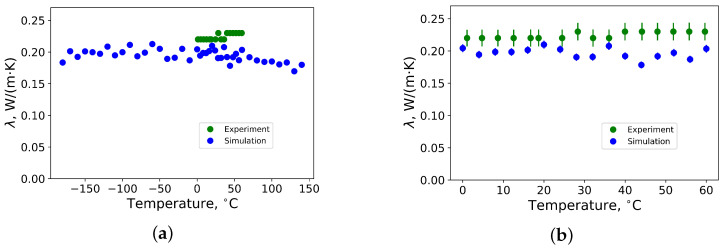
(**a**) Thermal conductivity of polybutadiene rubber vulcanized by 2.8 phr of sulfur in temperature range between −180 ${}^{\circ }$C and 140 ${}^{\circ }$C from MD simulations; (**b**) thermal conductivity of polybutadiene rubber vulcanized by 2.8 phr of sulfur in temperature range between 0 ${}^{\circ }$C and 60 ${}^{\circ }$C from MD simulations (blue) and measurements (green) by the Heat Flow Meter method.

For the sample of polybutadiene rubber crosslinked by 2.8 phr of sulfur, the same tendency is observed, where thermal conductivity above room temperature remains almost constant or slightly decreases (see Figure 7). It has a global maximum of approximately −50 ${}^{\circ }$C degrees, which is also close to the experimental value (see Figure 4).

**Figure 8:**
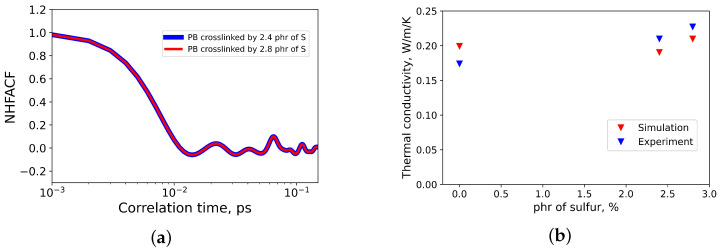
(**a**) The first minima of normalized heat flux autocorrelation functions (NHFACF) of polybutadiene crosslinked by 2.4 and 2.8 phr of sulfur; (**b**) thermal conductivities of polybutadiene rubbers crosslinked by different amounts of sulfur.

In Ref. [24], a simple harmonic oscillator model has been proposed for the estimation of the frequency of phonon modes, which are dominant in heat transfer. According to the model, the first minima of the heat flux autocorrelation functions are connected with the frequency of these modes. Applying this result to the normalized heat flux autocorrelation functions (NHFACF) of rubbers (see Figure 8a), one can see that the first minimum of NHFACF is located roughly at 15 fs, which corresponds to wave number of $\bar{\nu }\approx 353\;{\mathrm{cm}}^{-1}$. This is close to the wave number of C-C-C deformations [35]. As a result, the dominant means of heat transport in rubber is governed by deformations of polymeric chains. A similar heat transport mechanism was observed in polyethylene and polyisoprene rubbers [24,36].

Combining this with the results taken from Refs. [22,26], it was revealed that the thermal conductivity of polybutadiene rubber increases with an increase in sulfur content (see Figure 8b). The same tendency was observed in experiments [37] and in MD simulations of polyisoprene and polybutadiene rubbers [26].

## 4. Conclusions

The thermal conductivities of polybutadiene rubbers vulcanized by 2.4 and 2.8 phr of sulfur were calculated by MD simulations and measured by the heat flow meter method in the temperature range from 0 ${}^{\circ }$C to 60 ${}^{\circ }$C at atmospheric pressure. Results from MD simulations are in good agreement with experimental data. From an analysis of the normalized heat flux autocorrelation function, it was found that the main mechanism of heat transfer in these rubbers is cased by the transport of low-frequency phonons, which are cased by deformations of polymeric chains. The tested force field is sufficient for the prediction of thermal conductivities of polybutadiene rubbers and, for an estimation, their glass transition temperatures. Finally, it can be concluded that MD simulations using the Green–Kubo approach can be used to determine the thermal conductivities of rubbers as a function of temperature for their macro-scale modeling by FEM.

## Figures and Tables

**Table 1:** Rubber and chemicals used to prepare samples.

Ingredient	Quantity (phr)
Liquid butadiene rubber (LBR)	100
Zinc Oxide	3
Stearic acid	2
TBzTD	1
CBS	1.5
Sulfur	2.4 and 2.8

## Data Availability

Not applicable.

## Abbreviations

The following abbreviations are used in this manuscript:

FEM (finite element method), phr (parts per hundred rubber), *NPT* (isothermal–isobaric ensemble), *NVT* (canonical ensemble), *NVE* (microcanonical ensemble), DSC (dynamic scanning calorimetry), DMA (dynamic strain amplitude), NHFACF (normalized heat flux autocorrelation function).
